# Analgesic effect of single-shot ropivacaine at different layers of the surgical site in primary total hip arthroplasty: a randomised, controlled, observer-blinded study

**DOI:** 10.1186/s13018-020-02182-8

**Published:** 2021-01-22

**Authors:** Qiang Xiao, Bing Xu, Haoyang Wang, Zhenyu Luo, Mingcheng Yuan, Zongke Zhou, Fuxing Pei

**Affiliations:** 1 Department of Orthopedics, Chengdu Second People’s Hospital, Chengdu, People’s Republic of China; 2 Department of Orthopedics, West China Hospital/West China School of Medicine, Sichuan University, 37# Wuhou Guoxue Road, Chengdu, 610041 People’s Republic of China

**Keywords:** Total hip arthroplasty, Local infiltration anaesthesia, Postoperative pain

## Abstract

**Objectives:**

The aim of this study was to evaluate the efficacy of local infiltration anaesthesia (LIA) during primary total hip arthroplasty (THA) via a posterolateral approach under general anaesthesia and to compare the efficacy of LIA in all layers with LIA in the deep and superficial fascia.

**Patients and methods:**

One hundred twenty patients were randomised into three groups: LIA in the deep and superficial fascia (group A), LIA in all layers (group B) and the control (group C). The primary outcomes were the visual analogue scale (VAS) pain scores at rest and on movement within 72 h (h) postoperatively. The secondary outcomes included opioid consumption, patient satisfaction, range of motion (ROM), straight leg raise completion rate, length of hospital stay, opioid-related side effects and wound complications. We followed the patients until 6 months after discharge.

**Results:**

At 2 and 6 h, groups A and B had lower resting VAS scores than group C (*p* < 0.01); at 12 h, group B had a lower resting VAS score than group C (*p* < 0.05). At 6 and 12 h, the movement VAS scores in groups A and B were lower than those in group C (*p* < 0.01). Groups A and B had similar VAS scores during the observation period. Groups A and B had higher levels of patient satisfaction than group C (*p* = 0.03 and *p* = 0.018, respectively). Opioid consumption was similar in the three groups. There were no significant differences in the other secondary outcomes amongst the three groups. No difference was found in hip rehabilitation or chronic pain during the follow-up period.

**Conclusion:**

Single-shot LIA with ropivacaine alone reduces the pain score during the first 12 postoperative hours and improves patients’ satisfaction with THA. LIA in the deep and superficial fascia and LIA in all layers have similar analgesic effects. LIA in the deep and superficial fascia may be an alternative method to LIA in all layers.

## Introduction

Postoperative pain after total hip arthroplasty (THA) can increase the risk of postoperative complications, length of hospital stay and cost; thus, it influences patients’ satisfaction [1]. In China, approximately 8% of patients are not satisfied with THA, and pain is the most important factor influencing that dissatisfaction [2]. Multimodal analgesia regimens are widely used to manage perioperative pain for THA, and local infiltration anaesthesia (LIA) is one of the main components [1]. The LIA technique used during THA was first introduced by Kerr and Kohan [3], who used a “cocktail” that was a mixture of ropivacaine, ketorolac acid and adrenaline to achieve local infiltration in all layers at the surgical site and an indwelling catheter in the wound for the continuous injection of analgesics after the operation. After that, several randomised controlled trials (RCTs) on single-shot LIA in THA were conducted; the outcomes of these RCTs were controversial, and the local analgesics (LAs) used in these RCTs were not all the same [4–14]. Some of these studies [6, 8, 10–13] used cocktails of drugs for the infiltration, and other studies [9, 14] used single drugs. Some of these studies [6, 10, 12, 14] showed that single-shot LIA could reduce early postoperative pain and/or opioid use after THA, and other studies [8, 9, 11, 13] showed that single-shot LIA did not provide additional analgesic effects in THA managed by a multimodal analgesia regimen. Previous studies showed that a lateral femoral cutaneous nerve block could relieve the postoperative pain from a posterolateral approach THA and reduce the use of opioids after femoral neck fracture surgery performed through a lateral incision [15–17]. This suggests that LIA of the deep and superficial fascia at the surgical site may relieve postoperative pain after the posterolateral approach because the incision is located in the area of lateral femoral cutaneous nerve innervation.

Therefore, we performed an RCT to evaluate the efficacy of single-shot LIA with ropivacaine alone in primary THA performed via a posterolateral approach under general anaesthesia and to compare the efficacy of LIA in all layers with that of LIA in the deep and superficial fascia.

## Patients and methods

Prior to patient enrolment, this trial was registered in the Chinese Clinical Trial Registry (identifier ChiCTR1800016700, registration date 18 June 2018, http://www.chictr.org.cn/edit.aspx?pid=28384&htm=4) and was approved by the Ethics Committee and Institutional Review Board of West China Hospital, Sichuan University. Written informed consent was obtained from all patients before surgery. The study was conducted at the Department of Joint Surgery of West China Hospital, Sichuan University, according to the CONSORT (Consolidated Standards of Reporting Trials) Statement. The clinical study was performed in accordance with the Declaration of Helsinki on ethical principles for medical research involving human subjects.

We recruited consecutive patients aged 18-80 years with an American Society of Anesthesiologists Physical Status I–III who were scheduled for primary unilateral THA via a posterolateral approach under general anaesthesia. The exclusion criteria included a known allergy, contraindication to or dependence on drugs used in the protocol, a history of alcohol addiction, reoperation, severe liver and kidney dysfunction, pregnancy or breastfeeding, refusal to participate and failure to complete the related indicators.

Patients were randomly divided into three groups: LIA in the deep and superficial fascia (group A), LIA in all layers (group B) and control (group C). A random sequence was generated by a computer and hidden in consecutive numbered, sealed, opaque envelopes by research assistants who did not participate in the data analysis. The envelope was opened prior to surgery, and the LA was prepared by operating room nurses not involved with patient care or evaluation. Patients, anaesthesiologists, care providers, analgesic drug managers and data collectors were all blinded to the allocation sequence. The surgeon (Z-K. Z) who performed the LIA could not be blinded, but he was not involved in the data collection or postoperative management related to this trial.

All patients underwent standardised general anaesthesia. All THAs were performed by the same senior surgeon (Z-K. Z) using a posterolateral approach and a single brand of cementless components (DePuy Synthes). No postoperative drain was used. Group A and group B received infiltration performed by the surgeon (Z-K. Z) with 80 ml of 0.25% ropivacaine (Naropin, AstraZeneca) solution. The infiltration technique described by Kerr and Kohan was used [3], but we used single-shot infiltration. Group A received LIA of the deep and superficial fascia. After suturing the deep fascia, 40 ml of solution was infiltrated into the deep fascia, and the other 40 ml of solution was infiltrated into the superficial fascia. In group B, 40 ml of solution was infiltrated into the deep tissues, including the tissues around the rim of the acetabulum, external rotators and gluteus tendon. The remaining 40 ml of solution was infiltrated into the superficial tissues, including the fascia, subcutaneous tissues and skin. No LIA was performed in group C.

All patients received the same analgesic protocol. Patients received celecoxib (Celebrex, Pfizer) 200 mg orally every 12 h for pre-emptive analgesia after admission and celecoxib 200 mg orally every 12 h for postoperative pain. When the VAS score was ≥ 4 (visual analogue scale, 0 = no pain, 10 = worst pain) and ≤ 6, patients received oxycodone hydrochloride prolonged-release tablets (OxyContin, Mundipharma) 10 mg orally every 12 h as a rescue medicine. When the VAS score was > 6, a subcutaneous injection of 5 mg of morphine was administered immediately as a rescue medicine. After discharge, patients continued to receive 200 mg celecoxib orally every 12 h for 2 weeks, and then we used analgesics according to patient need. The remaining aspects of perioperative management were the same. All patients were treated with 1.5 g cefuroxime before the skin incision and then 1.5 g every 8 h after the operation for 24 h. All patients were given 2000 Axa IU of enoxaparin sodium (Clexane, Sanofi) 6 h after the operation, and 4000 Axa IU was given daily from the first day after the operation until discharge. After discharge, 10 mg of rivaroxaban (Xarelto, Bayer) was given once a day for 10 days. The rehabilitation nurses guided the patients to perform active ROM exercises and strength training postoperatively, and the patients began walking with crutches on the first day after the operation. The discharge criteria mainly included functional recovery and pain relief: the ability to walk more than 30 m with crutches, the ability to climb stairs, the ability to dress, the ability to go to the bathroom independently, a VAS score lower than 3 at rest and a VAS score lower than 5 during movement.

The primary outcome was the VAS pain score at rest and during movement. The resting VAS score was assessed at 2, 6, 12, 24, 36, 48, 60 and 72 h after the operation, and the movement VAS score was assessed at 6, 12, 24, 36, 48, 60 and 72 h after the operation.

The secondary outcomes included opioid consumption, patient satisfaction, ROM of the hip on postoperative day 2, the number of patients completing the straight leg raise at postoperative day 1, the length of hospital stay, opioid-related side effects (nausea and vomiting, urinary retention and pruritus) and wound complications. In addition, we followed up with the patients at 2 weeks, 3 months and 6 months after the operation to assess hip pain, hip function, wound complications and quality of life. Opioid consumption was uniformly converted to equivalents of oral morphine for the statistical analysis. The conversion factor was as follows: 10 mg of subcutaneously injected morphine was equal to 30 mg of oral morphine, and 10 mg of oral OxyContin was equal to 20 mg of oral morphine [18, 19].

### Statistical analyses

Continuous data are shown as the means and standard deviations (SDs). Categorical data are presented as numbers and percentages. One-way analysis of variance (ANOVA) with post hoc Bonferroni tests were used for normally distributed continuous variables, and the Kruskal-Wallis test with the post hoc Nemenyi test were used for skewed continuous variables. Chi-square or Fisher tests were used for categorical variables. Statistical significance was set at *p* < 0.05. Statistical analyses were performed with SPSS (version 22.0; IBM).

The sample size was calculated by the G Power Version 3.1.9 (Franz Faul; Uni Kiel, Germany) software. A pilot study was performed with 15 patients (5 in each group), and the resting VAS scores in the three groups at 6 postoperative hours were 2.2, 2.0 and 2.6, with a SD was 0.704, and then the effect size *f* = 0.35 was calculated. According to this effect size, an alpha level (2 tailed) = 0.05, and power = 0.95, 35 patients would be needed in each arm of the study using a fixed effect one-way ANOVA design. Assuming a 15% loss to follow-up, 40 patients were enrolled in each arm.

## Results

**Fig. 1:**
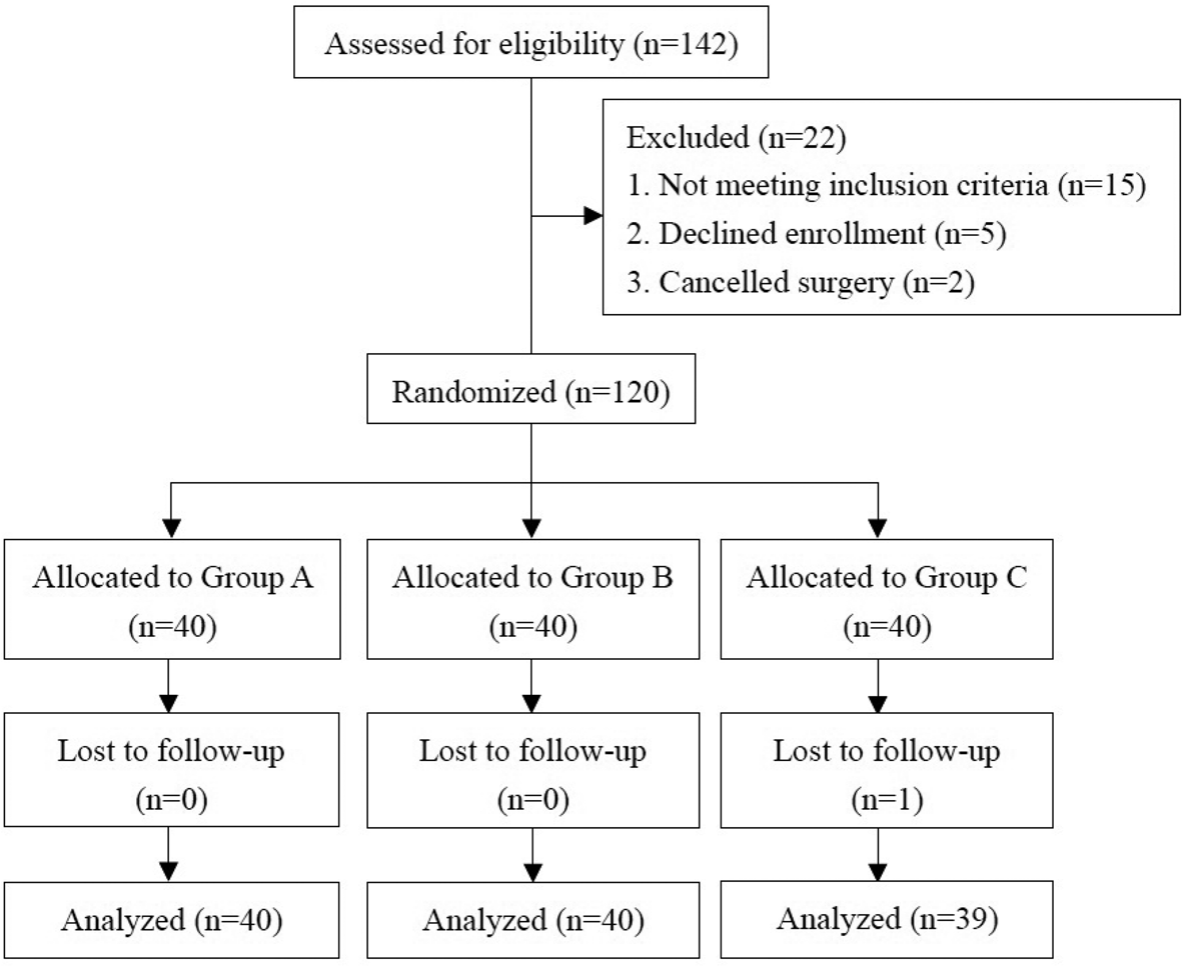
CONSORT (Consolidated Standards of Reporting Trials) flow diagram. Group A, LIA in the deep and superficial fascia; group B, LIA in all layers, group C, the control

From July 2018 to December 2018, a total of 142 patients scheduled for primary unilateral THA were assessed for eligibility. Twenty-two patients were excluded: 15 patients did not meet the inclusion criteria, 5 patients declined to participate and 2 patients had their surgeries cancelled. Therefore, there were 120 patients randomised to the three groups. One patient in group C died 2 weeks after discharge due to a cause (pulmonary hypertension) unrelated to THA. The remaining 119 patients completed the trial (Fig. 1). No significant differences were identified amongst the groups with respect to the patients’ baseline demographic variables and perioperative characteristics (Table 1).

**Table 1:** Demographic variables and perioperative characteristics

Variables	Group A (***n*** = 40)	Group B (***n*** = 40)	Group C (***n*** = 39)	***P*** values^†^
Age (years)	56.0 ± 13.2	55.2 ± 15.2	56.4 ± 13.6	0.926
Sex, M/F	22/18	27/13	16/23	0.061
Operative side, R/L	27/13	24/16	23/16	0.693
Height (cm)	160.6 ± 8.7	161.6 ± 7.5	160.3 ± 8.5	0.762
Weight (kg)	61.9 ± 12.3	60.2 ± 11.3	60.8 ± 9.9	0.784
BMI (kg/m^2^)	23.9 ± 4.0	22.9 ± 3.1	23.6 ± 2.9	0.403
ASA score I/II/III	3/29/8	2/25/13	6/27/6	0.170
HHS	52.6 ± 14.0	48.9 ± 10.1	51.1 ± 13.0	0.423
HOOS				0.842
Symptoms	10.4 ± 2.7	9.7 ± 2.5	9.9 ± 2.8	0.453
Pain	20.1 ± 5.1	19.2 ± 5.0	19.9 ± 5.2	0.731
Daily living	30.4 ± 6.3	29.0 ± 6.3	28.7 ± 7.2	0.647
Sports and recreational activities	5.3 ± 1.3	5.0 ± 1.3	5.6 ± 1.6	0.177
Quality of life	5.0 ± 1.8	4.6 ± 1.6	4.9 ± 1.7	0.568
Movement VAS	4.1 ± 1.3	4.4 ± 0.7	4.5 ± 0.9	0.176
Resting VAS	0.7 ± 0.7	0.7 ± 0.6	0.7 ± 0.5	0.967
SF-12				
PCS	14.8 ± 2.8	13.8 ± 2.6	14.4 ± 2.2	0.195
MCS	21.4 ± 2.9	21.1 ± 1.6	22.2 ± 1.6	0.053
Duration of surgery (min)	63.3 ± 18.2	66.8 ± 21.0	62.8 ± 19.1	0.615

Values are the mean ± SD or count as appropriate

*M* male; *F* female; *R* right; *L* left; *BMI* body mass index; *HHS* Harris Hip Score; *ASA* American Society of Anesthesiologists; *HOOS* Hip Disability and Osteoarthritis Outcome score; *VAS* visual analogue scale; *SF-12* the 12-Item Short Form Health Survey; *PCS* physical component summary; *MCS* mental component summary

^†^The *p* value represents the result of one-way analysis of variance for independent means for continuous variables or the chi-square test for independent proportions amongst the 3 groups

At 2 and 6 postoperative hours, the resting VAS scores in groups A and B were significantly lower than those in group C (*p* < 0.01, and *p* < 0.001, respectively), with no significant differences between groups A and B; at 12 postoperative hours, the VAS score in group B was significantly lower than that in group C (*p* < 0.05), with no significant difference between groups A and B or groups A and C (*p* > 0.05) (Tables 2, 3).

**Table 2:** VAS scores and morphine equivalents consumption

Variables	Group A (***n*** = 40)	Group B (***n*** = 40)	Group C (***n*** = 39)	***P*** values*
Resting VAS				
2 h	2.4 ± 0.7	2.3 ± 0.9	3.4 ± 1.0	< 0.001**
6 h	2.3 ± 0.6	2.1 ± 0.6	2.9 ± 0.7	< 0.001**
12 h	2.2 ± 0.8	2.0 ± 0.5	2.6 ± 0.8	0.002**
24 h	1.8 ± 0.4	1.8 ± 0.6	1.8 ± 0.5	0.834
36 h	1.7 ± 0.6	1.6 ± 0.6	1.6 ± 0.6	0.850
48 h	1.2 ± 0.4	1.2 ± 0.4	1.3 ± 0.6	0.268
60 h^#^	1.1 ± 0.3	1.1 ± 0.3	1.3 ± 0.5	0.153
72 h^#^	1.1 ± 0.3	1.0 ± 0.2	1.2 ± 0.4	0.122
Movement VAS				
6 h	3.8 ± 0.7	3.7 ± 0.9	4.6 ± 0.8	< 0.001**
12 h	3.7 ± 0.8	3.5 ± 0.6	4.2 ± 0.9	< 0.001**
24 h	3.3 ± 0.5	3.2 ± 0.5	3.4 ± 0.5	0.244
36 h	3.2 ± 0.5	3.1 ± 0.4	3.1 ± 0.5	0.474
48 h	2.7 ± 0.6	2.8 ± 0.4	2.9 ± 0.8	0.464
60 h^#^	2.8 ± 0.6	2.6 ± 0.5	2.9 ± 0.3	0.170
72 h^#^	2.1 ± 0.3	2.2 ± 0.4	2.3 ± 0.5	0.317
Morphine equivalents (mg)				
0-12 h	2.3 ± 5.4	1.9 ± 5.0	4.2 ± 7.7	0.191
0-24 h	9.1 ± 13.3	9.6 ± 14.6	11.8 ± 15.7	0.688
Total during hospitalisation	32.5 ± 47.2	32.1 ± 48.2	48.6 ± 57.8	0.270

Values are the mean ± SD

*From one-way analysis of variance or Kruskal-Wallis analysis for independent means for continuous variables amongst the three groups

**Significant

^#^25/26/23 patients in group A/B/C were in the hospital at 60 postoperative hours, and 24/26/23 patients in group A/B/C were in the hospital at 72 postoperative hours

**Table 3:** VAS scores post hoc test

Variables	Group A (***n*** = 40)	Group B (***n*** = 40)	Group C (***n*** = 39)	***P*** values^**✚**^			
				***P***	***P***1	***P***2	***P***3
Resting VAS							
2 h	2.4 ± 0.7	2.3 ± 0.9	3.4 ± 1.0	< 0.001^✚✚^	1.000	< 0.001^✚✚^	< 0.001^✚✚^
6 h	2.3 ± 0.6	2.1 ± 0.6	2.9 ± 0.7	< 0.001^✚✚^	0.937	< 0.001^✚✚^	< 0.001^✚✚^
12 h	2.2 ± 0.8	2.0 ± 0.5	2.6 ± 0.8	0.002^✚✚^	0.482	0.075	0.001^✚✚^
Movement VAS							
6 h	3.8 ± 0.7	3.7 ± 0.9	4.6 ± 0.8	< 0.001^✚✚^	1.000	< 0.001^✚✚^	< 0.001^✚✚^
12 h	3.7 ± 0.8	3.5 ± 0.6	4.2 ± 0.9	< 0.001^✚✚^	0.722	0.007^✚✚^	< 0.001^✚✚^

Values are the mean ± SD

^✚^From one-way analysis of variance for independent means for continuous variables amongst the three groups. P values from analysis with use of the post hoc Bonferroni test; *P p* value of group A vs B vs C; *P1 p* value of group A vs B; *P2 p* value of group A vs C; *P3 p* value of group B vs C

^✚✚^Significant

Similarly, at 6 and 12 postoperative hours, the movement VAS scores in groups A and B were significantly lower than those in group C (*p* < 0.01, and *p* < 0.001, respectively), with no significant differences between groups A and B (*p* > 0.05) (Tables 2, 3). At the rest of the time points during the study period, no significant differences were found in resting and movement VAS scores amongst the three groups (Table 2).

Groups A and B had higher levels of patient satisfaction with pain control than group C (*p* = 0.03, and *p* = 0.018, respectively), and more patients in groups A and B than in group C could complete the straight leg raise test at postoperative day 1 (*p* = 0.004, and *p* = 0.032, respectively) (Table 4). The amounts of morphine equivalent consumption at all time points as well as total consumption in groups A and B were less than those in group C, although the differences were not significant (Table 2). Moreover, there were no significant differences in the ROM of the hip at postoperative day 2, length of hospital stay, opioid-related side effects and wound complications amongst the three groups (Tables 4, 5).

**Table 4:** Postoperative outcomes

Variables	Group A (***n*** = 40)	Group B (***n*** = 40)	Group C (***n*** = 39)	***P*** values^**△**^			
				***P***	***P***1	***P***2	***P***3
Hospital stay (days)	2.9 ± 1.0	2.9 ± 0.8	2.8 ± 0.9	0.869			
Pain satisfaction (*n*)							
Very satisfied	25	26	15	0.033^**△△**^	0.816	0.033^**△△**^	0.018^**△△**^
Somewhat satisfied	15	14	24	0.033^**△△**^	0.816	0.033^**△△**^	0.018^**△△**^
Somewhat dissatisfied	0	0	0				
Very dissatisfied	0	0	0				
Function satisfaction (*n*)							
Very satisfied	18	19	18	0.975	0.823	0.918	0.905
Somewhat satisfied	22	21	21	0.975	0.823	0.918	0.905
Somewhat dissatisfied	0	0	0				
Very dissatisfied	0	0	0				
Straight leg raise (*n*)	31	28	18	0.010^**△△**^	0.446	0.004^**△△**^	0.032^**△△**^
Range of motion (°)							
Flexion	106.0 ± 5.8	104.3 ± 6.3	103.5 ± 6.4	0.176			
Abduction	33.3 ± 3.7	32.9 ± 3.4	33.5 ± 3.8	0.768			
Extension	0	0	0				

Values are the mean ± SD or count as appropriate

^**△**^From one-way analysis of variance for independent means for continuous variables or the chi-square test for independent proportions amongst the three groups. *P p* value of group A vs B vs C; *P1 p* value of group A vs B; *P2 p* value of group A vs C; *P3 p* value of group B vs C

^**△△**^Significant

**Table 5:** Complications

Variables	Group A (***n*** = 40)	Group B (***n*** = 40)	Group C (***n*** = 39)	***P*** values^**※**^
Nausea and vomiting	1	2	1	1.000
Urinary retention	2	1	0	1.000
Pruritus	0	0	0	
Aseptic fat liquefaction of wound	2	2	1	1.000
Superficial infection	0	0	0	
Deep infection	0	0	0	
Haematoma	0	0	0	
Superficial wound necrosis	0	0	0	

Values are counts

^**※**^Chi-square or Fisher’s test for independent proportions amongst the three groups

During the 6-month follow-up period after THA, resting and movement VAS scores, Harris Hip Scores (HHS), Hip Disability and Osteoarthritis Outcome scores (HOOS) and the 12-Item Short Form Health Survey (SF-12) results amongst the three groups were not significantly different. Only 2 patients in group C continued to take analgesic drugs 2 weeks after discharge, and no patients complained about hip pain at the last follow-up. No patient experienced wound complications during the follow-up period (Table 6).

**Table 6:** Follow-up outcomes

Variables	Group A (***n*** = 40)	Group B (***n*** = 40)	Group C (***n*** = 39)	***P*** values^**▲**^
Resting VAS				
2 weeks	0.2 ± 0.4	0.2 ± 0.4	0.3 ± 0.4	0.839
3 months	0.1 ± 0.3	0.1 ± 0.3	0.1 ± 0.3	0.999
6 months	0.1 ± 0.3	0.1 ± 0.2	0.1 ± 0.3	0.704
Movement VAS				
2 weeks	1.2 ± 0.4	1.2 ± 0.4	1.3 ± 0.4	0.932
3 months	0.7 ± 0.7	0.7 ± 0.6	0.7 ± 0.7	0.991
6 months	0.6 ± 0.6	0.5 ± 0.6	0.6 ± 0.6	0.578
HOOS-3 months				
Symptoms	16.6 ± 1.2	16.3 ± 1.1	16.1 ± 1.1	0.244
Pain	31.1 ± 1.6	30.9 ± 1.5	30.5 ± 1.7	0.238
Daily living	60.9 ± 1.7	61.3 ± 1.9	60.7 ± 1.7	0.420
Sports and recreational activities	12.0 ± 1.5	12.5 ± 1.4	11.9 ± 1.4	0.153
Quality of life	11.8 ± 1.3	12.0 ± 1.3	11.7 ± 1.2	0.455
HHS-3 months	89.1 ± 2.2	88.7 ± 2.3	88.2 ± 2.1	0.213
SF-12-3 months				
PCS	22.3 ± 1.1	21.8 ± 1.3	22.0 ± 1.2	0.274
MCS	26.1 ± 1.3	26.0 ± 1.3	25.9 ± 1.4	0.737
HOOS-6 months				
Symptoms	16.9 ± 0.9	17.1 ± 0.8	16.9 ± 0.8	0.467
Pain	34.2 ± 1.8	33.9 ± 1.4	33.6 ± 1.3	0.336
Daily living	63.5 ± 1.4	63.1 ± 1.7	63.1 ± 1.5	0.396
Sports and recreational activities	13.0 ± 1.0	13.0 ± 1.1	12.7 ± 1.1	0.301
Quality of life	13.0 ± 1.1	12.9 ± 1.0	12.6 ± 0.7	0.193
HHS-6 months	92.1 ± 1.9	92.6 ± 2.2	92.0 ± 2.0	0.285
SF-12-6 months				
PCS	23.2 ± 1.1	23.3 ± 1.0	23.0 ± 0.8	0.291
MCS	27.0 ± 0.9	27.4 ± 1.1	27.0 ± 0.9	0.092

Values are the mean ± SD

*HHS* Harris Hip Score; *HOOS* Hip Disability and Osteoarthritis Outcome; *PCS* physical component summary; *MCS* mental component summary

^**▲**^From one-way analysis of variance for independent means for continuous variables amongst the three groups

## Discussion

In our study, with the LIA method and analgesic protocol we used, the two LIA groups had less pain than the control group within 12 h after primary THA and had a higher level of patient satisfaction. Furthermore, despite the lack of a significant difference, the total morphine equivalents consumed in groups A and B were 16.1 mg (33.1%) and 16.5 mg (34.0%) less than that consumed in group C, respectively. In addition, the two LIA groups had comparable analgesic effects.

In previous RCTs on single-shot LIA in THA [6, 8–14, 20], there was a lack of consensus on the dosage, volume and constituents of LA, and there was controversy regarding whether single-shot LIA had a role in the multimodal analgesia regimen for THA (Table 7). Three of these RCTs used a single LA for infiltration, two of which used ropivacaine and one of which used levobupivacaine [9, 14, 20]. Zoric et al. demonstrated that 80 ml of 0.2% ropivacaine used for LIA did not reduce opioid consumption and did not improve postoperative rehabilitation after THA [9]. In contrast, Murphy et al. showed that using 60 ml of 0.25% levobupivacaine for LIA in THA significantly reduced morphine consumption by 45.8% in the first 12 h after surgery [14]. In our study, we used 80 ml of 0.25% ropivacaine for single-shot LIA because this dose was close to the dose authorised for infiltration in China. Our findings showed that single-shot LIA with ropivacaine alone was effective for elective primary THA. Although single-shot LIA does not improve postoperative rehabilitation in patients undergoing primary THA, it can reduce pain within 12 h and reduce morphine consumption by more than 30% after THA.

**Table 7:** Relevant RCTs regarding single-shot LIA in total hip arthroplasty

Authors	Study design	No. of patients	LIA drugs	Conclusion
Busch et al. [10]	RCT	LIA: 32, control: 32	LIA (100 ml): 0.4% ropivacaine + 0.03% ketorolac + 0.6:100000 epinephrine + 0.005% morphine Control: not given LIA	LIA reduces early stage postoperative pain and opioid consumption after THA.
Liu et al. [6]	RCT	LIA: 40, control: 40	LIA (60 ml): 0.008% morphine + 0.05% ropivacaine + 1.7% betamethasone + 0.8:100000 epinephrine Control (60 ml): normal saline	LIA reduces postoperative pain and opioid consumption and improves postoperative rehabilitation after THA.
Murphy et al. [14]	RCT	LIA: 45, control: 46	LIA (60 ml): 0.25% levobupivacaine Control (60 ml): normal saline	LIA reduces opioid consumption after THA.
Villatte et al. [12]	RCT	LIA: 75, control: 75	LIA (100 ml):0.235% ropivacaine + 0.5:100000 epinephrine Control: not given LIA	LIA reduces postoperative pain and opioid consumption after THA.
Titman et al. [20]	RCT	LIA: 19, control: 16	LIA (150 ml): 0.2% ropivacaine Control (150 ml): normal saline	LIA reduces early-stage postoperative pain after THA.
Lunn et al. [13]	RCT	LIA: 60, control: 60	LIA (150 ml): 0.2% ropivacaine + 1:100000 epinephrine Control (150 ml): normal saline	LIA does not reduce postoperative pain or opioid consumption after THA.
Dobie et al. [11]	RCT	LIA:50, control:46	LIA (200 ml): 0.125% Levobupivacaine + 0.5:100000 epinephrine Control: not given LIA	LIA does not reduce postoperative pain and does not improve postoperative rehabilitation after THA.
Zoric et al. [9]	RCT	LIA: 27, control: 24	LIA (80 ml): 0.2% ropivacaine Control (80 ml): normal saline	LIA does not reduce opioid consumption and does not improve postoperative rehabilitation after THA.
Hofstad et al. [8]	RCT	LIA: 55, control: 54	LIA (150 ml): 0.2% ropivacaine + 0.3:100000 epinephrine Control (150 ml): normal saline	LIA does not reduce postoperative pain or opioid consumption after THA.
Current study (2019)	RCT	Group A: 40, group B: 40, group C: 39	Groups A and B (80 ml): 0.25% ropivacaine Group C: not given LIA	LIA reduces early-stage postoperative pain, and LIA in the deep and superficial fascia had a similar analgesic effect to LIA in all layers in THA. LIA seems to help reduce opioid consumption but does not improve postoperative rehabilitation after THA.

*RCT* Randomised controlled trial, *LIA* Local infiltration anaesthesia, *THA* Total hip arthroplasty; group A LIA in the deep and superficial fascia group; group B LIA in all layers group; group C control group

The LIA technique used in previous studies infiltrated all layers, including the tissues around the rim of the acetabulum, external rotators and gluteus tendon [3]. To the best of our knowledge, this study was the first to report the effect of LIA of the deep and superficial fascia in THA. Thybo et al. reported that a lateral femoral cutaneous nerve block could relieve postoperative pain due to THA via a posterolateral approach [21]. In addition, a lateral femoral cutaneous nerve block could also reduce the opioid consumption of patients undergoing femoral neck fracture surgery via a lateral incision [16, 17]. The incision for the posterolateral approach in THA was partially located in the lateral femoral cutaneous nerve distribution area. This suggested that LIA in the deep and superficial fascia might relieve postoperative pain in patients undergoing THA surgery. Moreover, our several years’ experience of applying LIA in the deep and superficial fascia in THA has demonstrated its effectiveness. In this systematic RCT, the outcomes showed that LIA in the deep and superficial fascia had a similar effect to LIA in all layers and relieved the early postoperative pain due to THA surgery via a posterolateral approach. As LIA in the deep and superficial fascia is easier to perform than LIA in all layers, it might be an alternative method.

In our study, the analgesic effects in the two LIA groups lasted 12 h after THA. Previous studies reported that the longest analgesic effect duration of single-shot LIA in THA was 48 h [14]. To prolong the analgesic effect of LIA after THA, three methods were employed. The first method was adding epinephrine to local anaesthetic. Titman et al. used 150 ml of 0.2% ropivacaine for single-shot LIA and found that it could relieve the postoperative pain due to THA in the first hour after surgery [20]. However, Lunn et al. found that single-shot LIA with 150 ml of a solution composed of 0.2% ropivacaine and 1:100,000 epinephrine had no effect on postoperative pain due to THA surgery [13]. Therefore, this method seems unreliable. The second method was inserting an indwelling catheter in the surgical site to facilitate the injection of local anaesthetic after the surgery. Bianconi et al. reported that injecting 0.2% ropivacaine at 5 ml/h via an indwelling catheter in the surgical site until 55 postoperative hours could prolong the analgesic effect of LIA to 72 h after total joint arthroplasty surgery, and the pharmacokinetic results of this study confirmed the safety of the method [22]. However, Bianconi et al. enrolled total knee arthroplasty and THA patients at the same time and did not analyse them independently. Aguirre et al. showed that this method could significantly decrease morphine consumption by 24.3 mg (34.9%) during the first 48 postoperative hours compared with the placebo [4]. However, Solovyova et al. demonstrated that the continuous infusion of 0.2% ropivacaine at 5 ml/h for 48 h after surgery provided no additional analgesic benefit in THA managed with a multimodal analgesia regimen. A similar conclusion was drawn in two other previous studies [5, 23]. Therefore, the effect of this method needs to be verified. The third method was infiltration with sustained-release anaesthetics. EXPAREL (DepoFoam Bupivacaine) used polycystic liposomes as a carrier of bupivacaine; these liposomes could continuously release bupivacaine 72 h after a single-dose infiltration [24]. A meta-analysis performed by Zhang et al. showed that EXPAREL had a lasting analgesic effect for up to 48 h after THA surgery when used for LIA. Accordingly, this method is promising, but the drug is not yet widely used in China, and more research is needed to confirm its effectiveness.

In our study, two patients in group C needed analgesic drugs for more than 2 weeks; nevertheless, the follow-up results at 6 months showed that LIA had no effect on the postoperative pain score and hip rehabilitation after THA surgery. Similarly, Zoric et al. found that there were no significant differences in rehabilitation progress and chronic pain between the LIA group and control group at 3 months and 1 year after THA surgery [9]. However, Aguirre et al. demonstrated that THA patients could still benefit from LIA for 3 months after surgery [4]. Further studies are needed to evaluate the long-term effect of LIA in THA.

Our study had several limitations. First, the surgeons were not blinded to the group assignment; however, the patients, care providers, analgesic drug managers and data collectors were blinded, and the surgeons did not participate in the data collection and postoperative management related to this trial. Second, all patients in this study received general anaesthesia. Spinal anaesthesia is associated with improved perioperative outcomes, although there is limited quantitative evidence [25]. Therefore, our findings may not be applicable to patients who received spinal anaesthesia. Third, this study was conducted in a single centre, and all operations were performed by the same surgeon, which might reduce the bias caused by different surgeons. However, the surgeon in this study had completed more than 3000 THAs and was proficient. Therefore, we are not sure whether surgical technique would affect the results.

In conclusion, in patients undergoing primary THA via the posterolateral approach under general anaesthesia, single-shot LIA reduces the pain score during the first 12 postoperative hours and improves patient satisfaction. Furthermore, LIA seems to help reduce opioid consumption. LIA did not have a clinical effect on rehabilitation of the hip after THA. LIA in the deep and superficial fascia and LIA in all layers have similar analgesic effects in elective THA. LIA in the deep and superficial fascia may be an alternative method to LIA in all layers as it is obviously easier to perform.

## Data Availability

The datasets used and/or analysed during the current study are available from the corresponding author on reasonable request.

## Abbreviations

THA: Total hip arthroplasty
LIA: Local infiltration anaesthesia
VAS: Visual analogue scale
ROM: Range of motion
HHS: Harris Hip scores
HOOS: Hip Disability and Osteoarthritis Outcome scores
SF-12: The 12-Item Short Form Health Survey
